# Functional Traits, Morphology, and Herbage Production of Vernalised and Non-Vernalised Chicory cv. Choice (*Cichorium intybus* L.) in Response to Defoliation Frequency and Height

**DOI:** 10.3390/plants9050611

**Published:** 2020-05-11

**Authors:** Mancoba C. Mangwe, Racheal H. Bryant, Cristian A. Moreno García, Thomas M.R. Maxwell, Pablo Gregorini

**Affiliations:** Faculty of Agriculture and Life Sciences, PO Box 85084, Lincoln University, Lincoln 7647, Christchurch, New Zealand; Racheal.Bryant@lincoln.ac.nz (R.H.B.); cristian.morenogarcia@lincolnuni.ac.nz (C.A.M.G.); tom.maxwell@lincoln.ac.nz (T.M.R.M.); Pablo.Gregorini@lincoln.ac.nz (P.G.)

**Keywords:** *Cichorium intybus*, pastoral systems, thermal time, vernalisation, herbage production

## Abstract

Chicory (*Cichorium intybus* L.) used in pastoral systems has the attributes required of a forage species to reduce animal urinary nitrogen loading to soil, increase milk production, and enhance milk fatty acid profile to improve pastoral farm systems for matching increasing global demand for dairy products and environmental standards of livestock systems. Greater adoption of chicory requires confidence in management decisions that can control risks to farm production, namely bolting after vernalisation or a decline in persistence of chicory swards, which have slowed its adoption in pastoral systems. We, therefore, measured functional traits, morphology and herbage production of chicory under irrigated field conditions before and after vernalisation in Canterbury, New Zealand. The experimental site was laid out in a complete randomized block design with four replications where two regrowth intervals and two defoliation heights were applied. Regrowth interval had a stronger influence over functional traits and herbage production than defoliation height, with more pronounced effects after vernalisation. Plants managed under shorter regrowth intervals had narrower roots with lower concentration of sugars than plants under longer intervals, which might compromise their longevity. In addition, plants managed under shorter intervals remained mostly vegetative with heavier and longer leaves, though with reduced photosynthetic capacity than those managed under longer intervals. The thermal time to initiate stem elongation in plants managed under longer intervals was ~274 growing degree-days, with a mean stem elongation rate increasing linearly at 1.4 ± 0.08 mm/growing degree-days. The key outcomes of this research quantify the growing degree-days to initiate stem elongation post vernalisation, which provides management directive for timing of defoliation of chicory in order to maintain feed quality for grazing livestock. Alternating frequent and infrequent defoliation regimes might be used to optimise vegetative growth, root reserves, and pasture persistence.

## 1. Introduction

The challenges of maintaining productivity while meeting regulations for improved environmental outcomes and reducing water usage in pasture-based systems in New Zealand relying on perennial ryegrass (*Lolium perenne* L.) and white clover (*Trifolium repens* L.) highlights the need to consider the role of alternative forages in facing these issues. Recent research has shown environmental benefits from forages based on a range of plant traits including winter activity for reduced nitrate leaching [1]; high moisture content to reduce urinary nitrogen load [2]; low fibre content to reduce methane [3], and increased soil water use to reduce drainage [4,5]. Forage herbs such as chicory (*Cichorium intybus* L.) possess many of the attributes required to improve pastoral farm systems. Grasslands Choice is one of the two newer varieties of chicory available for commercial use on pastoral systems [6]. Grasslands Choice was bred from ‘Grasslands Puna’ chicory with selection emphasis on lower levels of sesquiterpene lactones, a compound that causes a taint or bitter aftertaste in the milk [7]. Chicory (cv. Choice) fed to dairy cows has shown to increase water intake and reduce urinary nitrogen concentration [8] in addition to supporting greater milk production [9,10] and enhance milk fatty acids profile [11,12]. However, the adoption of alternative forages in different grazing systems requires confidence in the response to management decisions and needs to identify risks to production in different environments.

The development of the reproductive stem in winter vernalised chicory plants has slowed its adoption in pastoral systems [6,13]. Mature reproductive stems of perennial herbaceous plants decrease forage quality due to a lowered leaf-to-stem ratio within individual plants and the more structural carbohydrates in mature stems [14,15]. This change in plant biomass partitioning and chemical and structural composition reduces feeding value of the plant [16,17]. By contrast, mechanisms and factors that trigger and influence the development and elongation of reproductive stems in vernalised chicory have been poorly studied. Clapham et al. [18] investigated the development dynamics of vernalised chicory cv. Puna in relation to thermal time and reported that time to bolting was 400 growing degree-days (GDD). Little is known about the development dynamics (i.e., bolting initiation and stem elongation) of the commercially available chicory cv. Choice. If the objective is to control the growth and development of the reproductive mature stems, chicory should be grazed before bolting to maintain plants in the vegetative state [19]. Understanding the morphological and physiological response to defoliation before bolting, i.e., 300 GDD or after bolting, i.e., 600 GDD and the traits associated with this is therefore strongly desirable to generate efficient and specific defoliation strategies of chicory pastures that could be used to control production and feeding value of chicory on farm. 

Another key limitation of chicory is limited persistence, though range in longevity has been reported in a review to be between 3 and 7 years [13], mainly dependent on suitability of climate, establishment and defoliation management. While there is a substantial volume of information available on the management options and associated impacts on forage yield of chicory in New Zealand and USA [6,20,21,22], there is poor information on forage production, morphology, and physiology of grazed chicory. Dairy cows have the potential to exacerbate treading impact and reduce plant density due to greater treading damage [23]. Additionally, most studies were conducted in rainfed environments without irrigation. In natural rain fed environments, where summer moisture deficits are common, chicory has shown to be more sensitive to defoliation interval than defoliation height [6,20,22]. The partitioning of photosynthates to above ground biomass of chicory is reduced under moisture deficit [24,25], so morphological response of chicory (to defoliation interval and/or height) may differ in an irrigated environment.

Morphological, physiological and functional traits have been useful to characterize plant species regarding their strategies to acquire, store, and invest nutrients and energy as well as to respond to variable frequency and height of defoliation [25,26,27]. The shoot size-shoot density dynamics have been particularly relevant to categorize plants according to their response to disturbance [28]. The shoot size-shoot density dynamic is determined by several major morphological traits such as leaf and stem mass, which are directly and indirectly associated to the overall plant fitness, persistence and herbage production [29]. The final number of leaves, leaf length, specific leaf area and overall absolute mass of leaves per plant are influenced by leaf appearance and elongation rates [30]. Leaf elongation is also important for maintaining the overall productivity of the plant and, therefore, the sward in general [31]. At species level, studies have shown that leaf elongation rate differs, depending on factors such as temperature and plant phenology stage; reproductive or vegetative [32,33]. To our knowledge, no studies have investigated the leaf elongation rate of Choice chicory in response to varying regrowth interval and at different phenological stages. 

Therefore, we conducted two experiments to examine the effects of defoliation regimes in irrigated dairy pastures on functional traits, morphology and herbage production of chicory, before and after vernalisation. The main aim of the first experiment was to compare the effects of two regrowth intervals, based on growing degree-days (GDD), and two defoliation heights on functional traits, morphology, and herbage production of vernalised and non-vernalised chicory cv. Choice. The second experiment measured the effects of the two regrowth intervals on leaf photosynthetic capacity and the development dynamics of leaves and stems.

## 2. Materials and Methods 

### 2.1. Site Description and Establishment of Chicory cv. Choice

Two experiments were undertaken at the Lincoln University Research Dairy Farm in Canterbury, New Zealand (43°38′ S, 172°28′ E; 17 m above sea level). The soil is classified as free-draining Templeton fine sandy loam soil with soil pH of 6.3 (1: 2.1 *v/v* soil–water slurry), Olsen phosphorus 30 mg/L, potassium 8.7, calcium 8.7, magnesium 0.19, and sodium 0.2 me/100 g as determined to 75 mm depth on 29 September, 2017. The experimental areas consisted of 10 × 40 m fenced areas within four larger 1.5 ha paddocks. Site preparation before planting involved spraying existing perennial ryegrass and white clover pastures with Weedmaster 540 (540 g/L glyphosate; Nufarm Limited, Auckland, NZ) at 2 litres/ha with a surfactant, Pulse™ at 100ml/100 litres of water on the 21 September, 2017. Paddocks were then chip-hoed and ploughed on 20 October, 2017, with chicory (cv. Choice) sown at 5.3 kg seed/ha on 17 November, 2017. No fertiliser was used during establishment. For post-emergence weed control, Preside^TM^ (800 g/kg flumetsulam; Dow Agroscience Limited, New Plymouth, NZ) was used after the emergence of the fourth leaf with the addition of a spraying oil at 500 ml/100 litres water.

The first defoliation occurred on 16 January 2018 when plants had at least seven fully developed leaves [34]. Defoliation was carried out by grazing with dairy cows for a maximum of three hours to minimize damage. The second defoliation occurred on 15 February, 2018, by grazing with dairy cows for several hours followed by mowing to a uniform height of 4 cm.

### 2.2. Management of Chicory (Experiment 1)

The experiment was conducted between 15 February 2018 and 5 February 2019 in pre-established plots of chicory cv. Choice. Treatments included two regrowth intervals based on the accumulated GDD, i.e., every 300 and 600 GDD, and two defoliation intensities at 4 and 8 cm stubble height. Treatments were applied in a completely randomized block design with four replications. In each of the four paddocks (blocks), defoliation regimes were randomly allocated to four 10 m × 10 m plots. Regrowth intervals in GDD were calculated above a 4 °C base temperature as:

GDD = ((daily maximum temperature + daily minimum temperature)/2) – 4))

The temperature was set to equal 4 °C when the mean daily temperature was below 4 °C [35]. Climatic data, including air temperature and rainfall were recorded at the NIWA weather station in Lincoln (Broadfield met station #17603), 2 km away from the experimental site. Monthly climate values during the experiment and a 10-year mean preceding the experiment are presented in Table 1. All plots were irrigated as necessary based on soil moisture data using the centre pivot system used by the farm. Monthly accumulated irrigation water applied during the experiment is presented in Table 1. Three applications of nitrogen fertiliser were applied as urea immediately after grazing all experimental plots in February, October, and December 2018 at doses of 30, 50, and 30 kg N/ha, respectively.

Grazing events were restricted to a maximum of 5 hours with 2 to 6 dairy cows (484 ± 16.9 kg live weight kg) to achieve the target defoliation height of each treatment. Cows were moved out of respective plots once the corresponding stubble height was reached based on a visual estimation. Cows were left for longer periods on the 4 cm treatments to ensure plots had significantly less leaf material than the 8 cm treatment plots [36]. Plots were not grazed during wet conditions or during winter to limit soil and plant damage and to improve plant survival [13]. During the experiment, the 300 GDD plots were grazed eight times with an average GDD of 326 ± 31 and 600 GDD plots were grazed five times with an average of 653 ± 32 GDD (Table 2). The first grazing for 300 GDD in March 2018 was delayed by two weeks (180 GDD) due to resources being committed to a separate experiment at the experimental site (Table 2). The first defoliation post vernalisation was due to occur in early September 2018 when accumulated thermal time was reached but was delayed until early October due to insufficient herbage. Defoliation in October was a combination of grazing followed by mowing to respective treatment heights (4 and 8 cm). 

### 2.3. Morphological and Physiological Measurements (Experiment 1)

For comparison of treatment effects on morphological and physiological traits, six random plants per plot replicate were measured on three occasions between February 2018 and February 2019; once before vernalisation in autumn 2018 (representing regrowth in February and April) and twice after vernalisation in spring 2018 and summer 2019 (spring regrowth between October and November, and summer regrowth December to February). Each plant was dug from the ground and separated into roots, shoots (primary and secondary), alive leaves and dead material (leaf plus stem dead material). Primary shoots were defined as those growing from the crown while secondary shoots were defined as the lateral shoots on the primary shoots [21]. The roots were washed and the taproot diameter (mm) of each plant was measured at the top widest part of the taproot. Due to difficulties in digging the whole taproot intact, only the top 15 cm of the taproot was washed and oven dried at 60 °C for 48 h to determine dry weight and water soluble carbohydrates (WSC) content [37]. Aboveground herbage was oven dried separately at 60 °C and dry weight was recorded.

### 2.4. Herbage Production Measurements (Experiment 1) 

Pre- and post-herbage mass was determined by harvesting to ground level all herbage within three 0.25-m^2^ quadrats before and after each defoliation event (3 × 4 reps = 12 quadrats per treatment). Accumulated herbage mass was calculated as the difference between pre-graze mass of current grazing event and post-graze mass of previous grazing event. Plant density in each plot was determined once, before vernalisation (autumn 2018) and twice after vernalisation (spring 2018 and summer 2019) by counting the number of plants within a 0.25-m^2^ quadrat immediately after a grazing event.

### 2.5. Plant Function of Vernalised Chicory Plants (Experiment 2)

Plant measurements (recorded every 2 to 3 days) commenced immediately after the final defoliation in Experiment 1 (5 February, 2019) and ceased after 475 GDD (17 March, 2019), when branching of selected main stems was too prolific for accurate record of measurements. For this study, we used plots from the 4 cm stubble height treatment with the corresponding regrowth intervals of 300 GDD and 600 GDD. Six plants per plot (*n* = 24 plants per treatment) were randomly selected and one primary shoot randomly selected from each plant was marked with coloured wire for monitoring of plant functional traits. To ensure consistency among treatments, any selected plants exceeding 4 cm height were cut to the target treatment height using scissors. 

The number of visible leaf tips, length of each leaf (from the base up to the tip of the leaf), length of visible stem, and onset of senescence (decrease in green length) were recorded. Leaves were counted acropetally, with the first leaf identified as leaf 1. These measurements enabled us to estimate the number of leaves on the primary shoot over time, elongation rate of each leaf, time to bolting (stem appearance), stem elongation rate and time to senescence of old leaves. Thermal times (e.g., degree-days to first leaf appearance after defoliation, initiation of stem elongation, beginning of senescence) were determined when 50% or more of observed plants reached the aforementioned phenological stages. Leaf elongation rate (mm/GDD) was calculated as the ratio between the increase of leaf length and the accumulated GDD on two consecutive samplings.

At the end of the regrowth period, leaf chlorophyll content was measured from three reproductive plants of the 600 GDD plots and three vegetative plants of the 300 GDD plots using the Soil Plant Analysis Development (SPAD, Minolta Camera Co., Osaka, Japan) chlorophyll meter. From each selected shoot, three mature leaves were measured; we took three readings per leaf (at a third, a half and at two thirds of the distance from the base) and averaged to one value per leaf [38]. At the end of the experiment, plants holding selected shoots were dug out. Taproots were washed and their diameter measured (see Experiment 1). The number of live leaves and shoots per plant were recorded. Specific leaf area (cm^2^/g) was calculated as the ratio of plant leaf area (LI-COR leaf area meter LI-3100 Li-Cor, (Lincoln, NE, USA)), and the dry weight of all the leaves. All plant components were dried at 60 °C separately and weighed to determine their absolute dry mass.

### 2.6. Data Analysis 

All data were analysed using R Core Team. In Experiment 1, we used mixed effect models from ‘lme4’ package version 1.1–21 of R [39] where plot was used as the experimental unit, regrowth interval (300 GDD and 600 GDD), defoliation height (4 cm and 8 cm) and age (before and after vernalisation) were included as fixed effects and block and sampling day as nested random effects. The ‘emmeans’ package version 1.4.5 of R, using the Tukey method for means separation at significance of *p* < 0.05.

In Experiment 2, developmental parameters (leaf length, leaf and stem elongation rates) were modelled as functions of cumulative GDD using polynomial mixed model regressions with the ‘lmer’ function of the ‘lme4’ package version 1.1–21 of R. Block was included as a random effect in all models. Leaf length data were also modelled as a function of leaf number (leaf position) to determine length of longest leaf. Based on the mixed model regression equations, predicted values were generated and plotted using the ‘ggeffects’ package version 0.14.2 of R [40] Additionally, the derivative of the model was calculated in order to determine where the GDD, a local maximums, occurred in the respective regression equations. These values for GDD were then applied to the original regression to determine what the maximum values were. Modelled results are presented up to leaf number 7 for all treatments because leaf number eight and above in each shoot for 600 GDD plants had three or less observations during the experiment.

A principal component analysis (PCA) was used to summarize the variation of functional, morphological, and physiological traits of vernalised chicory plants under the two defoliation intervals. Dimensionality reduction analyses were conducted using the ‘factoextra’ package, version 1.0.7 of R [41], with defoliation intervals as explanatory variables and plant traits as response variables. 

## 3. Results

Overall, regrowth interval had the greatest effect on morphological traits and on herbage production followed by age (i.e., before and after vernalisation); defoliation height had no major effects on the aforementioned parameters (Table 3).

### 3.1. Morphology of Individual Chicory Plants

There was a regrowth interval by age interaction (*p* < 0.05) on the number of shoots and leaves per plant (Table 3) of chicory plants. Before vernalisation, chicory plants in all treatments had 1.4 ± 0.31 shoots per plant and remained in a rosette growth form (Table 4). There was no effect of treatment on leaves per plant (14.6 ± 2.11 leaves/plant) which exhibited large variation (range between 10 and 19 leaves/plant). After vernalisation, the number of shoots per plant increased (Table 4; *p* < 0.0001). For example, primary shoots were two-fold greater in 300 GDD plants than 600 GDD plants (*p* < 0.0001), while secondary shoots were approximately eight times greater for 600 GDD plants than 300 GDD plants (*p* < 0.0001). As a result, after vernalisation the total number of shoots per plant was 1.9 greater in 600 GDD plants than 300 GDD plants. Likewise, the number of leaves per plant increased after vernalisation, and were 19% greater for 600 GDD plants than 300 GDD plants, regardless of defoliation height.

There was an interaction (*p* < 0.001) between regrowth interval and age on absolute leaf and stem mass. Before vernalisation, absolute leaf mass was 57% greater for 600 GDD plants than 300 GDD plants (*p* < 0.01), regardless of defoliation height. In contrast, after vernalisation, absolute leaf mass was similar for all treatments. Plants exposed to a longer regrowth interval (600 GDD) had 2.5 times greater absolute stem mass than 300 GDD plants after vernalisation (*p* < 0.0001). As a result, total plant weight (leaf plus stem mass) was 1.4 times greater for 600 GDD plants than 300 GDD plants (Table 4). The seasonal changes in plant morphology are presented in Figures S1 and S2.

There were main effects of regrowth interval and age (*p* < 0.0001) on root WSC concentration (Table 3). The concentration of WSC in chicory roots was 10% and 33% greater for 600 GDD plants than 300 GDD plants before and after vernalisation, respectively, regardless of defoliation height. Chicory root WSC concentration declined by nearly 46% and 35% after vernalisation for 300 GDD and 600 GDD plants, respectively (Table 4). There were main effects of regrowth interval and age on root diameter (*p* < 0.0001), and a defoliation height by age effect on root diameter (*p* = 0.032). Before vernalisation, the root diameter of 600 GDD chicory plants grazed to 4 cm were not different from 300 GDD treatment plants. After vernalisation, root diameter of all 600 GDD plants was greater than 300 GDD plants, regardless of defoliation height. 

### 3.2. Herbage Production and Plant Density of Chicory Sward

Herbage mass and plant density for all treatments before and after vernalisation are shown in Table 5. Pre-graze mass was consistently greater (*p* < 0.05) for 600 GDD plants than 300 GDD plants throughout the growing seasons. On average, post-graze mass was ~20.4% and 17.8% greater for 8 cm than 4 cm stubble height before and after vernalisation, respectively, regardless of regrowth interval. Post-graze mass was ~63% greater (*p* < 0.0001) after vernalisation than before vernalisation. 

There was a regrowth interval by age interaction for accumulated herbage mass (*p* = 0.002). Before vernalisation, accumulated herbage mass was not different between treatments (4357 ± 213 kg/ha of dry matter (DM) between February and May 2018), while after vernalisation between October 2018 and February 2019, 600 GDD plants accumulated 12.8% greater herbage mass than 300 GDD plants, regardless of defoliation height. 

Plant density (plants/m^2^) declined (*p* < 0.0001) by nearly 45% after vernalisation and was not different (*p* ≥ 0.496) between defoliation regimes.

### 3.3. Functional Traits after Vernalisation 

**Figure 1:**
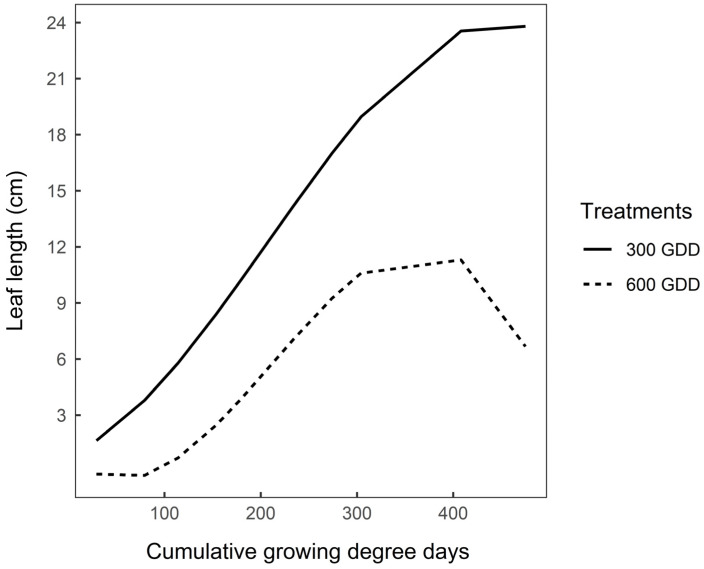
Modelled mean leaf length (cm) of the first seven leaves of 300 GDD and 600 GDD chicory plants during a regrowth between February and March 2019 (475 GDD). Lines are predicted values plotted against cumulative growing degree-days (GDD) using equations: y = 0.88 + 0.02x +0.00026x^2^ – 0.00000042x^3^ for 300 GDD plants and y = 0.88 – 0.049x + 0.00051x^2^ – 0.00000081x^3^ for 600 GDD plants (*n* = 24). Root mean square prediction error = 5.4, Error due to random effects (i.e., block) = 0.61.

**Figure 2:**
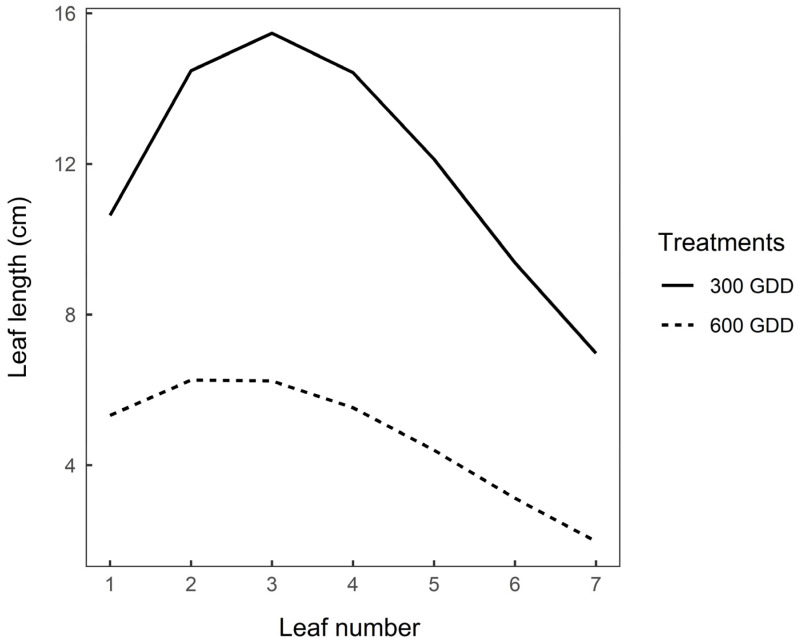
Modelled mean leaf length (cm) of each of the first seven leaves of 300 growing degree-days (GDD) and 600 GDD chicory plants during regrowth between February and March 2019 (475 GDD). Lines are predicted values plotted against leaf number (leaf position) using equations: y = 7.85 + 9.56x – 2.2x^2^ + 0.13x^3^ for 300 GDD plants and y = 4.4 + 2.87x – 0.75x^2^ + 0.05x^3^ for 600 GDD plants (*n* = 24). Root mean square prediction error = 7.6; error due to random effects (i.e., block) = 0.52.

**Figure 3:**
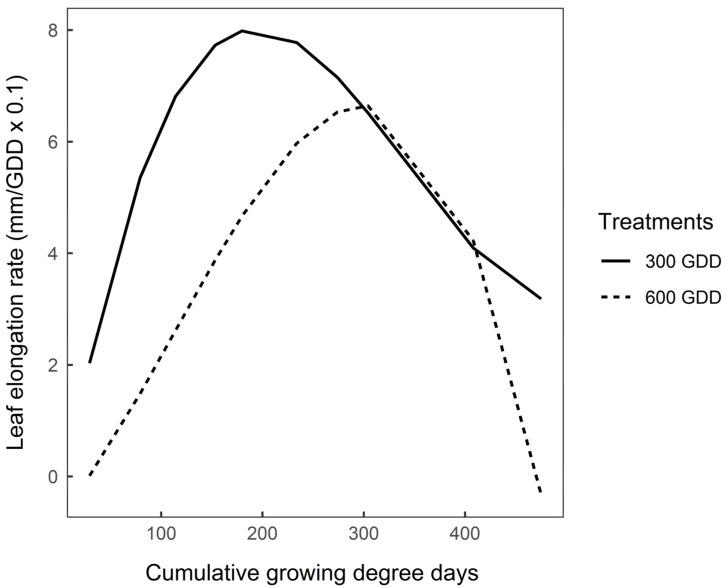
Modelled mean leaf elongation rate (mm/GDD × 0.1) of 300 GDD and 600 GDD chicory plants during a regrowth between February and March 2019 (475 GDD). GDD = Growing degree-days. Lines are predicted values plotted against cumulative growing degree-days (GDD) using equations: y = −0.69 + 0.1x − 0.00037x^2^ + 0.00000036x^3^ for 300 GDD plants and y = −0.69 + 0.02x + 0.0001x^2^ − 0.00000031x^3^ for 600 GDD plants (*n* = 24). Root mean square prediction error = 6.24; error due to random effects (i.e., block) = 0.52.

The effects of previous defoliation management on end of season (late summer/early autumn) leaf length and elongation rates of vegetative (300 GDD) and reproductive (600 GDD) plants are presented in Figure 1, Figure 2 and Figure 3. Following defoliation, leaves appeared sooner in 300 GDD plants than 600 GDD plants (39.1 vs. 55.5 GDD, respectively, *p* < 0.05). The relationship between leaf length and cumulative GDD was cubic (*p* < 0.0001) for both 300 GDD and 600 GDD plants (Figure 1), and the intercept of the predicted regression curve was similar for both treatments (*p* = 0.28). However, the mean leaf length was consistently greater for 300 GDD plants than 600 GDD plants (Figure 1; Figure 2; *p* < 0.05). Maximum length was achieved at third leaf for 300 GDD and at both second and third leaf for 600 GDD plants (Figure 2). 

Leaf elongation rate (mm/GDD) plotted against cumulative GDD also showed a cubic relationship (*p* < 0.001) for both 300 GDD and 600 GDD plants (Figure 3). Maximum leaf elongation rate in the 300 GDD plants was 0.80 mm/GDD compared with 0.66 mm/GDD for 600 GDD plants (Figure 3; *p* < 0.05). Leaves of 300 GDD plants reached maximum leaf elongation rate after 185 GDD while 600 GDD plants reached maximum elongation rate after 300 GDD during the regrowth period (Figure 3; *p* < 0.05). The actual values of leaf length and elongation rate are presented in Figures S3–S5.

Sixteen (67%) of the 24 selected plants from the 600 GDD plots developed stems, while the rest switched to a vegetative state. All 24 selected plants in the 300 GDD plots remained vegetative during the experiment. The time to initiate the elongation of stems was consistent for all replicates at ~274 GDD with a mean stem elongation rate increasing linearly at 1.4 ± 0.8 mm/GDD (*p* < 0.01). Secondary shoots formed along the primary stems at the first three leaf positions, with the first secondary stem visible after 359 GDD.

**Figure 4:**
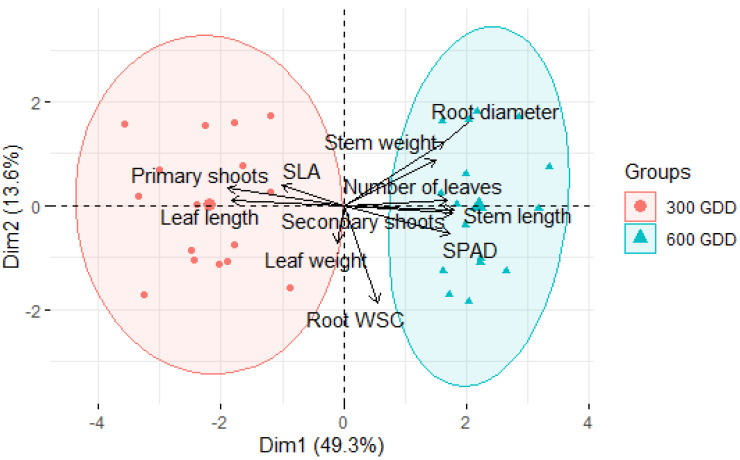
Principal Component Analysis biplot showing the variation of functional, morphological and physiological traits of vernalised chicory (cv. Choice) plants under the two defoliation intervals (300 and 600 GDD). WSC = water soluble carbohydrates; SLA = specific leaf area; SPAD = Soil Plant Analysis Development (SPAD) chlorophyll meter; GDD, growing degree-days; Dim1 = dimension 1; Dim2 = dimension 2.

An ordination diagram resulting from the dimensionality reduction analyses of the functional, morphological and physiological traits of vernalised chicory (cv. Choice) at the end of the regrowth period is presented in Figure 4. The first two principal components explained 62.9% of the total variability in the data. The ordination diagram revealed a major effect of defoliation interval, where samples of 300 GDD and 600 GDD plots are located at either side of the first axis. The longer defoliation interval was associated with a higher concentration of root WSC, more leaves and secondary shoots per plant, and a greater SPAD value, while the shorter interval was associated with more primary shoots, greater specific leaf area (SLA), and longer leaves (Figure 4). The mean values and the associated *p*-values of the measured traits are presented in Table 6.

## 4. Discussion

The regrowth intervals chosen in the current experiment were based on observations made by Clapham et al. [18], who reported that chicory cv. Puna bolted at around 400 GDD in southern Western Virginia (USA). With this in mind, we designed the experiment in such a way that plants of chicory cv. Choice would be defoliated to avoid (i.e., 300 GDD) or to encourage (i.e., 600 GDD) bolting, so we could quantify the impact of contrasting defoliation regimes on the responses of individual plants and of the plant community. Our results show interactions between defoliation regimes and the phenological stage of chicory plants, which created quite divergent morphological traits of leaves, shoots and roots and the overall herbage production. Consistent with our expectations, defoliation height had less of an effect (compared with regrowth interval) on most plant functional traits measured on chicory plants, nor on sward features such as plant density and herbage production as has been reported previously [6,21,22]. The lack of effect of defoliation height agrees with the observations of Lee et al. [26] who showed that although root reserves of non-structural carbohydrates were depleted following defoliation, they were replenished to pre defoliation levels within 310 GDD, with minimal influence from defoliation to 3 cm or 6 cm residual heights. Therefore, the following discussion is mainly focused on the effects of regrowth interval on morphology and plant functional traits of chicory and on the herbage production, before and after winter vernalisation.

### 4.1. Morphology and Functional Response of Chicory Plants

#### 4.1.1. Above Ground Plant Morphology of Non-Vernalised Chicory

The size and number of shoots per plant showed the greatest change over time and in response to defoliation regime. After establishment and before vernalisation, chicory plants in all treatments remained vegetative (no bolting stems) and displayed a similar plant architecture with one unique growing point and similar numbers of leaves per plant (Table 4). These similarities in plant architecture and phenological stage support previous results reported in New Zealand [21,42] and in USA [18,22]. In developing plants, leaves and roots compete for assimilates, with our results showing the greater sink strength for leaf development, and maintenance of root WSC. Little assimilate was used for shoot initiation which corresponds with Li et al. [21]. Collectively, these results confirm that the chicory cultivars Choice and Puna require vernalisation to initiate flowering, a process which occurs with low to mid temperatures between 0 °C and 12 °C during winter [43]. 

#### 4.1.2. Above Ground Plant Morphology of Vernalised Chicory Plants

Regardless of defoliation regime (i.e., regrowth interval and defoliation height), all plants increased the number of primary and secondary shoots after winter vernalisation. This response is consistent with Clapham et al. [18] and Li et al. [21], who revealed that the initial rosette of chicory split into multi-crowns during the second year on vernalised plants. The PCA results show the degree of morphological separation between the two defoliation frequencies (Figure 4). Frequently defoliated chicory plants (300 GDD) produced more primary shoots and of smaller size, while 600 GDD plants produced fewer but bigger primary shoots, which held more secondary shoots (Table 4). The primary shoots in 300 GDD plants were mostly vegetative material, while those formed on 600 GDD plants were mostly reproductive, with only occasional vegetative shoots. Li et al. [44] also reported a flush of vegetative shoots during the growing season in chicory plants grazed every 3–5 weeks, an interval equivalent to the 300 GDD interval in the current experiment. Following defoliation, plants produce more vegetative growth to increase their photosynthetic capacity and enhance growth rate to compensate for the lost biomass [45,46] and to replenish root reserves (Lee et al. [26]). There are two plausible explanations for the decreased number of primary shoots in 600 GDD plants; firstly, this can be due to an auxin-induced growth of a main stem that inhibits the outgrowth of lateral crown buds by diverting sugars away from the bud [47]. Frequent defoliation might remove the apical domination of the main stem and thus remove the growth inhibition of other crown buds allowing lateral crown shoots to be relatively further advanced before one shoot became dominant [48]. The other explanation is related to a shading effect, as young vegetative shoots of 600 GDD plants likely died because of lack of sufficient light to maintain growth as taller reproductive shoots capture most radiation [49,50]. Even though our results showed an increased photosynthetic capacity of leaves in 600 GDD plants (higher SPAD values), it might not have been sufficient to compensate for the reduced radiation interception by lateral shoots for sustaining growth which therefore resulted in premature senescence of lower leaves.

The increase in number of shoots after vernalisation was associated with the corresponding increased biomass of chicory plants after vernalisation (Table 4). When comparing the two regrowth intervals, individual plant biomass was greater for 600 GDD plants than 300 GDD plants. Generally, plants (grasses, legumes or herbs) that have reached reproductive stage are more productive in terms of individual plant biomass [15,21,51]. The increased plant weight for 600 GDD plants was related to stem weight. On a dry weight basis, stem accounted for 55% and 31% of the respective biomass for 600 GDD and 300 GDD plants after vernalisation. The proportion of stem observed here corroborates previous research showing a 15% to 50% range for chicory stem [18,21,42]. Similarly, for the vegetative 300 GDD plants, the mean plant weights (2.34 g and 4.86 g of DM per plant before and after vernalisation, respectively) are not dissimilar to the 3.86 g of DM per plant reported by Li et al. [21]. 

Despite the greater leaf number in 600 GDD plants after vernalisation, the absolute leaf mass per plant was not different between treatments (Table 4). This indicates the greater leaf weight of 300 GDD plants compared with 600 GDD plants, which is explained by longer leaves (Figure 1) with greater specific leaf area (Table 6) than the leaves of 600 GDD plants. Leaf size largely depends on plant phenology, with a progressive decrease in leaf size as the plant transitions from vegetative to reproductive stages [18,52]. As plants grow taller and prepare to reproduce, they partition most of their carbon to the development of stems (at the expense of leaves) to maintain an erect position to display their leaves and reproductive structures (flowers) within the well exposed layers of the canopy [15,53]. However, the leaf is the most important photosynthetic organ of plants and an important parameter in determining plant productivity [54]. Therefore, to cater for the increased plant demand, the small leaves from 600 GDD plants increased their photosynthetic capacity as shown by their higher SPAD values (Table 6). 

#### 4.1.3. Root Size and Concentration of Stored Carbohydrates 

The taproot of chicory is the main storage organ of WSC used for plant growth after defoliation or for winter dormancy [24,26]. Following defoliation, the leaf photosynthetic rate increases and the actively growing points in plants become high priority sinks of the currently produced photosynthetic carbon and the reserves located in the roots [46,55]. Consequently, the root size and concentrations of WSC tend to be lower in more frequently grazed plants of *Lolium perenne* L. than in less frequently grazed ones [51,56]. Our results showed that both the size of the roots and their concentration of WSC were lower for the 300 GDD plants than the 600 GDD plants before and after vernalisation. Similarly, Donaghy and Fulkerson [57] and Solomon et al. [51] have reported a decline in root WSC concentration from longer to shorter defoliation intervals in perennial and annual ryegrass plants, respectively. 

In addition to a regrowth interval effect, we also observed a phenological stage effect on the root WSC concentration. Vernalised plants had 46% and 35% lower concentration of root WSC than non-vernalised plants under 300 GDD and 600 GDD regrowth intervals, respectively. This is probably because chicory is a winter dormant plant, which utilises stored WCS for its survival during winter [13,21]. The plant is also transitioning to a reproductive stage after vernalisation, which requires greater energy resources [49]. 

### 4.2. Herbage Production and Plant Density of Chicory Swards

#### 4.2.1. Before Vernalisation

Before vernalisation between February and May 2018, all chicory treatments accumulated on average 6.4 ± 0.98 kg/ha DM herbage per GDD. Variation in accumulated herbage mass in response to contrasting regrowth intervals of the same forage species likely result from differences in plant density and plant size [28,51]. In the current experiment, no significant differences in plant density between treatments were found (Table 5). Additionally, all plants were in a rosette growth form with a similar plant structure, suggesting that 300 GDD chicory plants increased their growth rate after a grazing event to compensate for the lost biomass and subsequently accumulated equal yield as 600 GDD plants before vernalisation [45].

#### 4.2.2. After Vernalisation

After winter vernalisation, between October 2018 and February 2019, 600 GDD plants accumulated greater herbage mass than 300 GDD plants (8.7 vs. 7.7 ± 0.49 kg DM/ha per GDD). Several other studies have used a fixed number of calendar days or plant height as a criterion for when to defoliate chicory [6,20,22]. Regardless of which defoliation criterion was used in these studies, chicory plants defoliated after longer intervals accumulated greater herbage mass than those defoliated after shorter intervals. The total accumulated mass of vernalised chicory plants harvested every 300 GDD between October 2018 and February 2019 was consistent with the yield range of 9.6 t DM/ha – 11.2 t DM/ha reported for second year chicory defoliated at 3–5 weeks in spring and summer in New Zealand [6,20]. As plant density was not different between treatments, the differences among treatments in plant structure likely explains the differences in herbage production between the treatments after vernalisation. For example, 600 GDD plants had more stem material than 300 GDD plants. The rapid growth rate of the reproductive stem of vernalised 600 GDD plants (1.4 mm/GDD) might have increased the overall growth rate, subsequently accumulating more herbage mass than 300 GDD plants, which remained vegetative. Moreover, the greater concentration of WSC in roots of 600 GDD plants may explain their increased herbage mass compared with 300 GDD plants. Stored carbohydrates play a vital role in the growth and development of forages in temperate regions and their increased concentration is associated with greater forage yield [51]. 

After vernalisation, a decline in plant density of chicory is expected, regardless of defoliation regime [13,18,20]. With continuous defoliation, the root size and stored carbohydrates decrease, and the plants eventually die due to starvation, as has been shown for chicory and other temperate tap rooted forages such as red clover [21,58]. Another possible reason for the decline in plant density in chicory is treading damage of new buds on the crown and the crown itself, more especially when grazed by dairy cows [13]. Plants tend to increase their individual weight and that way, may compensate the loss of plants, maintaining forage mass [21,48], as was also observed in the current experiment. However, as Leach (1979) reported, “the inevitable loss of plants will subsequently decrease yields per unit area in the long run”. Under temperate conditions in New Zealand, a decline in chicory yield was observed after four years when the density was less than 25 plants/m^2^ [13].

### 4.3. Implications

In forages, there is a potential trade-off between herbage production and feeding value as plants exposed to longer regrowth intervals accumulate larger amounts of aerial mass, and reduce the palatable and the highly nutritious leaf proportion of the herbage [15]. In the current experiment, the time to the initiation of stem elongation for vernalised chicory cv. Choice was ~274 GDD. By grazing chicory every 300 GDD, we were able to control the growth and development of the mature stem material. Plants grazed at longer intervals (e.g., 600 GDD) had higher stem-to-leaf ratio suggesting that the herbage mass of such swards have reduced feeding value. However, our results showed that although 600 GDD plants produced more stem than leaf material, the absolute leaf mass did not differ from those plants grazed at 300 GDD intervals. This is consistent with the results by Clapham et al. [18], who also reported no differences in total leaf area between vegetative and reproductive plants of vernalised chicory. Therefore, appropriate herbage allocation of vernalised chicory herbage to livestock would need to account for stem refusal in reproductive plants, to avoid underfeeding and allow diet selection for digestible biomass to achieve high livestock performance. 

Finally, the consequence of low-quality mature stems on animal performance should be considered. Hunt and Hay [59] and McCoy et al. [60] reported that ruminants preferred plants that have not bolted. Barry [19] recommended that chicory should be grazed before bolting to maintain plants in the vegetative stage. Based on Barry [19] and the results from the current experiment, chicory plants should be grazed after a minimum of 4 weeks in autumn, and 3-4 weeks in spring and summer in order to control growth of reproductive stems. This period will also coincide with the peak leaf elongation rate (185–300 GDD; Figure 3), meaning grazing animals would capture the large, fully developed basal leaves of the plants before they senesce [18]. Extending the autumn regrowth interval similar to 6 weeks may aid recovery of root reserves, as seen in 600 GDD treatments and potentially improve plant longevity.

## 5. Conclusions

Under the conditions of the current experiment, plants of chicory cv. Choice managed under shorter regrowth intervals (i.e., 300 GDD) remained mostly vegetative with heavier and longer leaves, though with reduced photosynthetic capacity, than 600 GDD plants. Moreover, 300 GDD plants had narrower root diameter and lower root WSC, which is likely to compromise longevity of the forage crop. To maintain a vegetative, high feed quality forage crop after vernalisation, a more frequent defoliation regime is recommended, though the resulting decline in root WSC reserves is likely to compromise its longevity. Opportunity exists for a combination of frequent and infrequent defoliation regimes to optimise both vegetative growth and root reserves, and the effect of seasonal defoliation frequencies is an area for further exploration.

## Figures and Tables

**Table 1:** Monthly accumulated precipitation, irrigation water applied and mean air temperature in Lincoln, Canterbury, New Zealand during the experiment (Exp.) between February 2018 and March 2019. The 10-year means (Avg.) were calculated based on data from January 2008 to December 2017.

Month	Total Precipitation (mm)		Irrigation (mm)	Avg. Max Temp (°C)		Avg. Min Temp (°C)	
	During Exp.	10-yrs Mean	During Exp.	During Exp.	10-yrs Mean	During Exp.	10-yrs Mean
Feb-18	123	28.3	17.8	22.5	22.0	12.1	11.8
Mar-18	31.6	57.1	0.4	20.7	20.1	11.4	10.0
Apr-18	91.0	65.6	2.3	17.6	17.7	6.7	7.8
May-18	47.6	77.0	0	14.3	14.8	5.4	5.1
Jun-18	58.8	72.8	0	10.6	11.9	3.8	2.5
Jul-18	26.2	43.9	0	13.0	11.4	2.3	1.5
Aug-18	15.8	57.6	0	13.0	12.7	3.6	3.6
Sep-18	39.4	34.6	0	14.3	14.6	4.7	4.8
Oct-18	55.6	50.8	10.5	16.9	16.6	6.1	6.5
Nov-18	107.4	33.0	2.1	18.1	18.8	8.6	8.3
Dec-18	57.0	44.2	2.1	19.4	20.9	12.3	11.0
Jan-19	36.2	44.5	24.1	23.9	22.1	13.1	11.8
Feb-19	29.2	28.3	44.8	24.3	22.0	11.6	11.8
Mar-19	23.8	57.1	14.8	21.8	20.1	11.9	10.0
Total/Ave	742.6	694.8	118.9	17.9	17.6	8.1	7.6

**Table 2:** Defoliation dates and accumulated growing degree-days (GDD) between grazing events applied from February 2018 and January 2019.

Year	Harvest Period	Defoliation Intervals			
		300 GDD		600 GDD	
		Date	Accumulated GDD	Date	Accumulated GDD
2018	1	15-Feb		15-Feb	
	2	26-Mar	480		
	3	28-Apr	298 ^1^	6-Apr	609 ^1^
	4	5-Oct	740	5-Oct	907
	5	13-Nov	320 ^1^		
	6	13-Dec	298 ^1^	13-Dec	636 ^1^
2019	7	8-Jan	313 ^1^		
	8	5-Feb	402 ^1^	5-Feb	715 ^1^

^1^ Values used to calculate the average of accumulated GDD between grazing events for each treatment; 326 for 300 GDD and 653 for 600 GDD plots.

**Table 3:** Effects of regrowth interval, defoliation height and age, and interaction effects on morphological traits, plant density, and herbage production of chicory cv. Choice.

	Interval (I) ^1^	Height (H) ^1^	Age (A) ^1^	I × H	I × A	H × A	I × H × A
**At individual Plant Level**							
Number of leaves	0.006	0.777	<0.0001	0.319	0.048	0.728	0.351
Number of primary shoots	<0.001	0.996	<0.001	0.706	<0.001	0.281	0.304
Number of secondary shoots	<0.001	0.894	<0.001	0.240	0.001	0.796	0.240
Total shoots per plant	<0.001	0.748	<0.0001	0.193	<0.001	0.423	0.308
Absolute leaf mass (g DM ^2^)	<0.001	0.275	0.294	0.965	<0.001	0.249	0.233
Absolute stem mass (g DM)	<0.001	0.091	<0.001	0.571	<0.001	0.091	0.571
Total plant weight (g DM)	<0.001	0.465	<0.001	0.652	0.073	0.484	0.841
Root diameter (mm)	<0.001	0.203	<0.001	0.032	0.764	0.911	0.858
Roots WSC ^3^ (g/kg DM)	<0.001	0.149	<0.001	0.356	0.211	0.86	0.77
**At Pasture Level**							
Pre graze mass (kg/ha DM)	<0.001	0.522	0.678	0.600	0.566	0.580	0.536
Post graze mass (kg/ha DM)	0.961	0.014	<0.001	0.798	0.033	0.434	0.306
Accumulated herbage mass	<0.001	0.646	<0.001	0.467	0.002	0.771	0.724
Plant density (plants/m ^2^)	0.778	0.496	<0.001	0.695	0.643	0.343	0.253

^1^ Interval = 300 and 600 growing degree-days; Height = 4 and 8 cm; Age = before vernalisation (February–May 2018) and after vernalisation (October 2018–February 2019); ^2^ Dry matter; ^3^ Water soluble carbohydrates.

**Table 4:** Morphology of individual plants of chicory cv. Choice under contrasting defoliation regimes before and after vernalisation.

Interval	300 GDD ^1^		600 GDD ^1^		SEM ^2^
Height	4 cm	8 cm	4 cm	8 cm	
**Before Vernalisation (February–May 2018)**					
Number of leaves (leaves/plant)	15.2	13.8	13.9	15.4	2.11
Number of primary shoots (shoots/plant)	1.58	1.25	1.33	1.25	0.31
Number of secondary shoots (shoots/plant)	0	0	0	0	
Total shoots per plant (shoots/plant)	1.58	1.25	1.33	1.25	0.7
Absolute leaf mass (g DM)	2.44 ^b^	2.24 ^b^	3.57 ^a^	3.78 ^a^	0.26
Absolute stem mass (g DM)	0	0	0	0	
Total plant weight (g DM)	2.44 ^b^	2.24 ^b^	3.57 ^a^	3.78 ^a^	0.51
Root diameter (mm)	13.4 ^b^	14.7 ^b^	16.5 ^b^	18.8 ^a^	0.83
Roots water soluble carbohydrates (g/kg DM)	756 ^b^	766 ^b^	803 ^a^	870 ^a^	38.1
**After Vernalisation (October 2018–February 2019)**					
Number of leaves (leaves/plant)	31.6 ^b^	29.9 ^b^	38.3 ^a^	35.0 ^a^	1.49
Number of primary shoots (shoots/plant)	3.99 ^a^	4.46 ^a^	2.19 ^b^	2.12 ^b^	0.22
Number of secondary shoots (shoots/plant)	1.38 ^b^	0.38 ^b^	6.97 ^a^	8.53 ^a^	0.62
Total shoots per plant (shoots/plant)	5.37 ^b^	4.84 ^b^	9.16 ^a^	10.7 ^a^	0.51
Absolute leaf mass (g DM)	3.53	3.19	3.01	2.98	0.18
Absolute stem mass (g DM)	1.23 ^b^	1.77 ^b^	3.22 ^a^	4.30 ^a^	0.27
Total plant weight (g DM)	4.76 ^b^	4.96 ^b^	6.23 ^a^	7.28 ^a^	0.39
Root diameter (mm)	18.1 ^c^	19.0 ^c^	22.8 ^b^	26.8 ^a^	1.30
Roots water soluble carbohydrates (g/kg DM)	402 ^b^	417 ^b^	522 ^a^	568 ^a^	38.1

^a–c^ Means with different superscript letters differ significantly (*p* < 0.05); ^1^ GDD = Growing degree-days; ^2^ SEM = Standard error of the mean.

**Table 5:** Plant density and herbage production of chicory stands under contrasting defoliation regimes before and after vernalisation.

Interval	300 GDD ^1^		600 GDD ^1^		SEM ^3^
Height	4 cm	8 cm	4 cm	8 cm	
**Before vernalisation (February–May 2018)**					
Pre graze mass (kg/ha DM)	3510 ^b^	3293 ^b^	4648 ^a^	5032 ^a^	415
Post graze mass (kg/ha DM)	627	774	586	687	111
Accumulated herbage mass (kg/ha DM)	4192	4242	4637	4358	213
Plant density (plants/m^2^)	127	124	130.5	122	8.25
**After vernalisation (October 2018–February 2019)**					
Pre graze mass (kg/ha DM)	3510 ^b^	3659 ^b^	5248 ^a^	5407 ^a^	293
Post graze mass (kg/ha DM)	944 ^c^	1077 ^b^	973 ^c^	1182 ^a^	78.8
Accumulated herbage mass (kg/ha DM)	10,284 ^b^	10,316 ^b^	11,659 ^a^	11,576 ^a^	320
Plant density (plants/m^2^) ^2^	73	71	65	68	6.36

^a–c^ Means with different superscript letters differ significantly (*p* < 0.05); ^1^ GDD = Growing degree-days; ^2^ Values derived from summer measurements; ^3^ SEM = Standard error of the mean.

**Table 6:** Number of leaves and shoots per plant, absolute weight of above ground material, root diameter, SPAD and specific leaf area (SLA) of 300 GDD and 600 GDD plants of chicory cv. Choice destructively harvested after a regrowth between February and March 2019.

Item	600 GDD ^1^	300 GDD ^1^	SEM ^5^	*p*-Value
Number of live leaves per plant	42.6	28.2	4.13	0.019
Number of primary shoots per plant	2.10	3.79	0.34	0.011
Number of secondary shoots per plant ^3^	8.40 ± 0.4	-	-	-
Absolute leaf mass per plant (g DM^2^)	2.36	3.05	0.56	0.269
Absolute stem mass (g DM^2^) ^3^	2.99 ± 1.2	-	-	-
Final stem lenght (mm) ^3^	370 ± 160	-	-	-
Absolute dead material mass (g DM^2^)	0.87	0.75	0.11	0.213
Root diameter (mm)	21.4	19.1	1.22	0.272
SPAD ^4^	37.5	29.2	1.41	<0.001
Specific leaf area (cm^2^/g)	18.6	29.3	2.63	0.007

^1^ GDD = Growing degree-days; ^2^ DM = Dry matter; ^3^ Data are displayed as arithmetic means ± standard deviation. ^4^ Soil Plant Analysis Development (SPAD) chlorophyll meter. ^5^ SEM = Standard error of the mean.

## Supplementary Materials

The following are available online at https://www.mdpi.com/2223-7747/9/5/611/s1, Figure S1: Number of leaves and shoots per plants of chicory as influenced by grazing interval and season. Results are averaged over the grazing height treatments. Error bars are standard error of the mean. Figure S2: Absolute mass of above ground plant material of chicory as influenced by grazing interval and season. Results are averaged over the grazing height treatments. Error bars are standard error of the mean. Figure S3: Leaf length (cm) of 300 GDD and 600 GDD chicory plants during a regrowth between February and March 2019 (475 GDD). Lines are average values during each observation period. Shaded areas represent 95% confidence interval. Figure S4: Leaf length (cm) of the first seven leaves of 300 GDD and 600 GDD chicory plants during a regrowth between February and March 2019 (475 GDD). Lines are average values during each observation period. Shaded areas represent 95% confidence interval. Figure S5: Leaf elongation rate (mm/GDD × 0.1) of 300 GDD and 600 GDD chicory plants during a regrowth between February and March 2019 (475 GDD). Lines are average values during each observation period. Shaded areas represent 95% confidence interval.
