# Supplementary material for: Functional Traits, Morphology, and Herbage Production of Vernalised and Non-Vernalised Chicory cv. Choice (Cichorium intybus L.) in Response to Defoliation Frequency and Height
Source: Plants (Basel). 2020 May 11;9(5):611. doi: 10.3390/plants9050611 (PMC7285047; doi:10.3390/plants9050611)
Supplement: Supplementary file 1 [file plants-09-00611-s001.pdf]

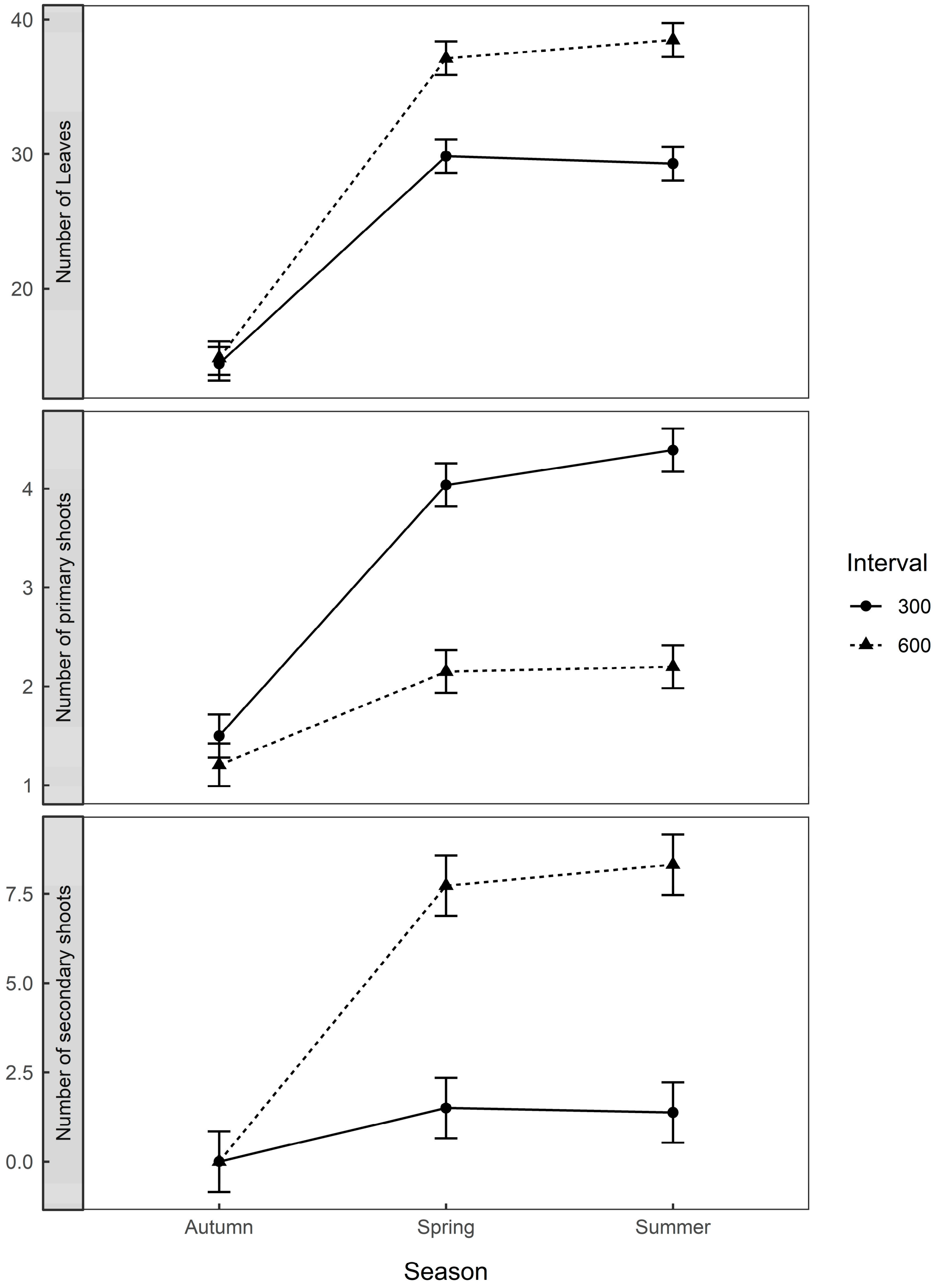

Figure S1. Number of leaves and shoots per plants of chicory as influenced by grazing interval and season. Results are averaged over the levels of height. Error bars are standard error of the mean.

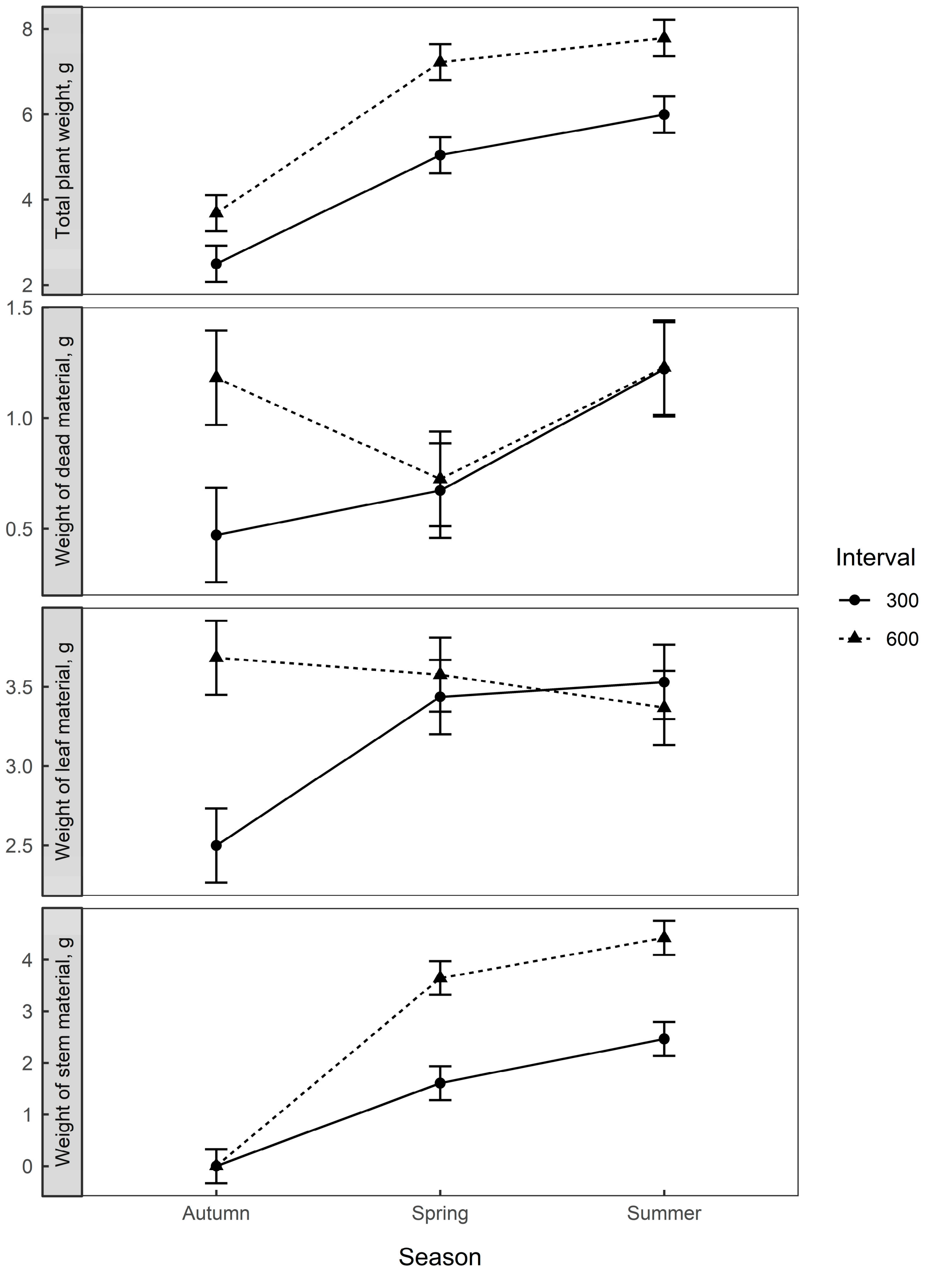

Figure S2. Absolute mass of above ground plant material of chicory as influenced by grazing interval and season. Results are averaged over the levels of height. Error bars are standard error of the mean.

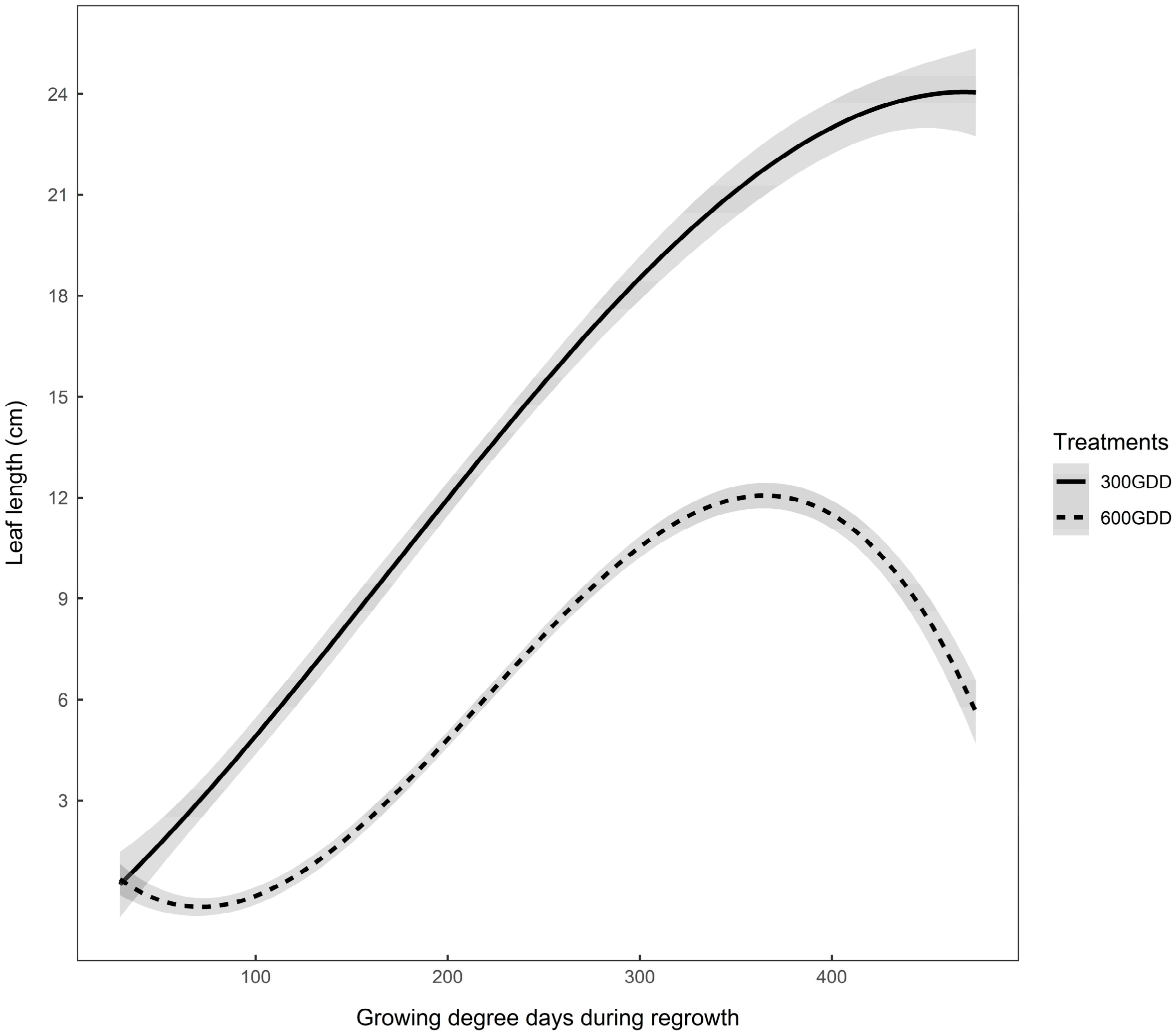

Figure S3. Leaf length (cm) of 300GDD and 600GDD chicory plants during a regrowth between February and March 2019 (475GDD). Lines are average values during each observation period. Shaded areas represent 95% confidence interval.

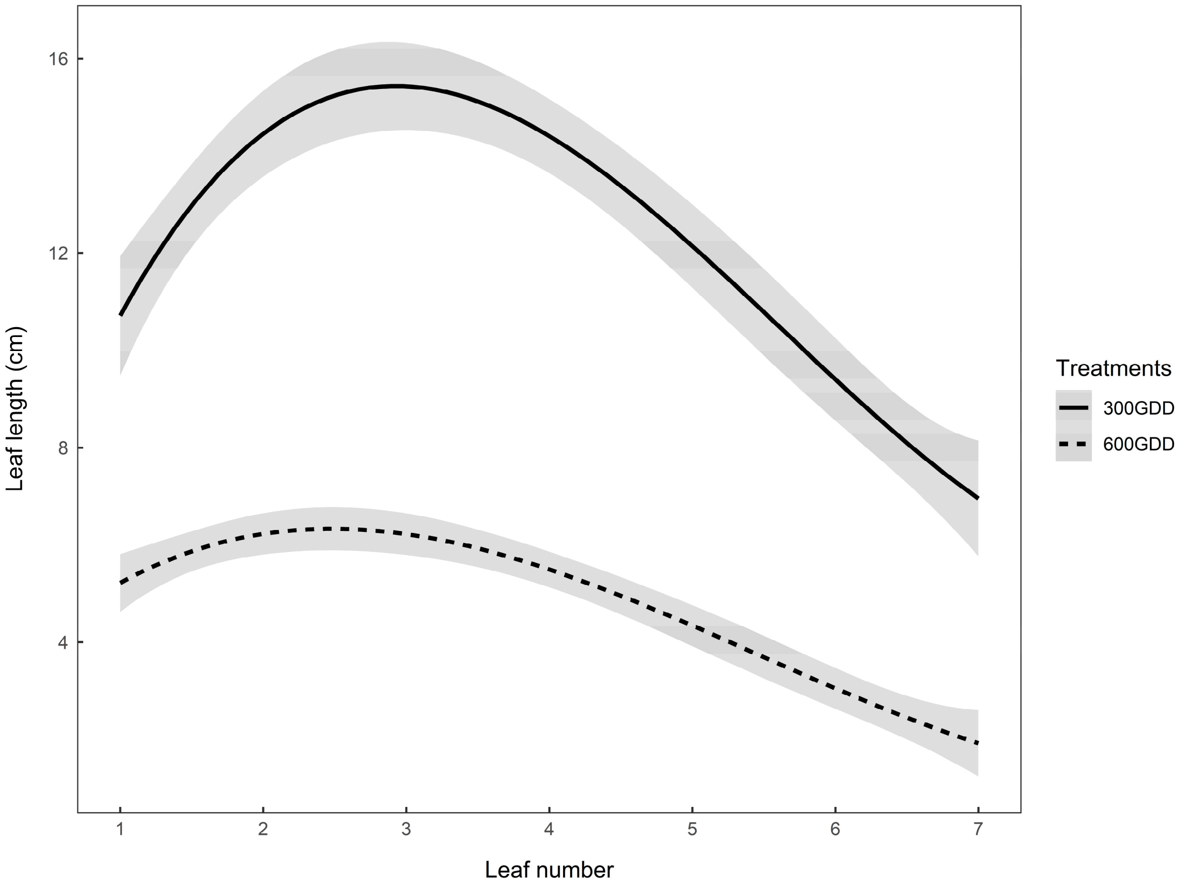

Figure S4. Leaf length (cm) of the first seven leaves of 300GDD and 600GDD chicory plants during a regrowth between February and March 2019 (475GDD). Lines are average values during each observation period. Shaded areas represent 95% confidence interval.

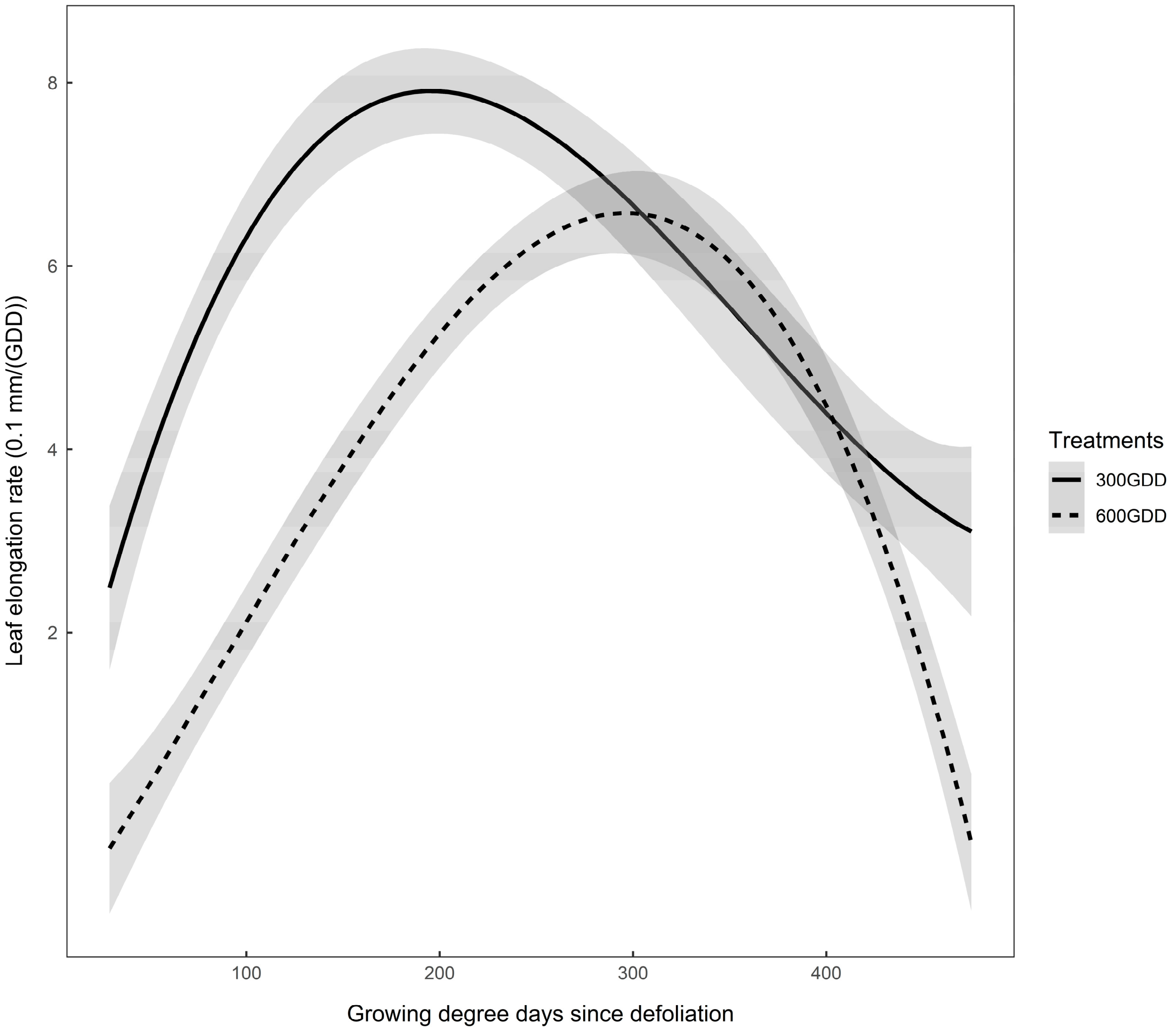

Figure S5. Leaf elongation rate (mm/GDD × 0.1) of 300GDD and 600GDD chicory plants during a regrowth between February and March 2019 (475GDD). Lines are average values during each observation period. Shaded areas represent 95% confidence interval.
